# Supplementary material for: IL-18 production is required for the generation of a Th1 response during experimental chromoblastomycosis
Source: PLoS One. 2025 May 2;20(5):e0322127. doi: 10.1371/journal.pone.0322127 (PMC12047800; doi:10.1371/journal.pone.0322127)
Supplement: Attachment — Submitted filename: Letter review.pdf [file pone.0322127.s002.pdf]

UNIVERSIDADE DE SÃO PAULO
Faculdade de Ciências Farmacêuticas
Departamento de Análises Clínicas e Toxicológicas

São Paulo, Feb 6, 2025

Editor PlosOne

Dear Editor,

Please find enclosed the manuscript entitled "**IL-18 production is required for the generation of a Th1 response during experimental chromoblastomycosis**", which we re-submit for publication.

We deeply thank the reviewer for the efforts spent in reading our manuscript. As follows, we addressed our replies for the comments. We also highlighted the changes within the manuscript.

Financial Support: This work was supported by São Paulo Research Foundation (FAPESP): Grant FAPESP 2016/04729-3. The funders had no role in study design, data collection and analysis, decision to publish, or preparation of the manuscript.

Av. Prof. Lineu Prestes, 580, Bloco 17
05508-000-São Paulo, SP – Brasil
(11) 3091-3661
dact@usp.br

UNIVERSIDADE DE SÃO PAULO
Faculdade de Ciências Farmacêuticas
Departamento de Análises Clínicas e Toxicológicas

Reviewer #1:

Methodology:
1-"Please include a subitem showing the macrophage obtention from BMDM.
correct this statement: intraperitoneal (i.p.) injection of 1 ml of PBS without (the control) or with 2 x 107
It seems that it is unclear to the reader if all experiments were conducted on all types of animals."

Thank you for the suggestion. This statement was improved in the new version of MS.

2-Results:
The first result does not mention the mice strain that the macrophages came from as well as the other results.
A better results description showing the mice strain used for macrophages assay is important. The graphs need a better description and indication of the cells used from mice strain and the correct pharmacological approach used indicating with or without inhibitor used.
At the present form important results description is missing.

Thank you for the observation. We include the mice strain in all assays

Reviewer #2:

1-The title of the paper should be modified to IL-18 production is required for the generation of a Th1 response during experimental chromoblastomycosis.

Thank you for the suggestion. We modified the title as recommend

2-The methods are appropriate to achieve the proposed objectives. However, the comparative evaluation of fungal quantification was obtained by counting colony-forming units (CFU) in macerates, liver, and spleen. I suggest that the authors also provide information about the aspects and compare the inflammatory reactions in the liver and spleen of both groups of animals.

Av. Prof. Lineu Prestes, 580, Bloco 17 (11) 3091-3661
05508-000-São Paulo, SP – Brasil dact@usp.br

UNIVERSIDADE DE SÃO PAULO
Faculdade de Ciências Farmacêuticas
Departamento de Análises Clínicas e Toxicológicas

Thank you for the suggestion. We realized the histopathology of organs, but we did not observe a significant difference, so we decided not to include this analysis

3-Authors must explain what they understand by severe forms of chromoblastomycosis (see page 3, second line of the penultimate paragraph and page 9 - second and third lines of the first paragraph) since in human disease this mycosis usually affects the skin and subcutaneous tissue and rarely manifests as a systemic disease. Insert the reference(s) in the first paragraph of page 9.

Thank you for your observation. We clarified in the new version of MS the information about the classification of severity of chromoblastomycosis

4-Insert the reference of the several papers mentioned in the last paragraph of page 9.

We added the references

5-Page 15 - Figure 2 caption - Why do the authors classify it as deep chromoblastomycosis? In this model, there is visceral involvement (liver and spleen), therefore systemic disease.

Thank you for your observation. We change the word deep by systemic

6-In the conclusion section, the authors should state that their work demonstrates, for the first time, the involvement of immune mechanisms mediated by the NRLP3 inflammasome in experimental chromoblastomycosis.

There was already evidence in the literature of the involvement of the NRLP3 inflammasome in chromoblastomycosis. We demonstrated for the first time that this inflammasome was involved in the production of IL-18 and activation of the Th1 response.

Reviewer #3:

1-The last part of page 8, and first topic on page 9 fit better in discussion. In this last one, when considered IFN-gamma, which patients? Is it part of the present research or, maybe it could fit better in discussion?

Av. Prof. Lineu Prestes, 580, Bloco 17
05508-000-São Paulo, SP – Brasil
(11) 3091-3661
dact@usp.br

UNIVERSIDADE DE SÃO PAULO
Faculdade de Ciências Farmacêuticas
Departamento de Análises Clínicas e Toxicológicas

Thank you for the suggestion. The paragraph commented on was moved to the discussion. Concerning IFN-g, we talk about a mild form of chromoblastomycosis

2-Page 18, second paragraph, correct NLRP3, not nlrp.

The word was corrected

3-How could authors explain the similar expression of IL10 and IL12?

At certain moments in the evolution of the disease, as in chromoblastomycosis, there is no cytokine predominance; thus, it is possible to observe both cytokines being secreted in a mixed system of cytokine-producing cells.

Sincerely Yours

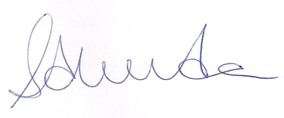

Dr. Sandro Rogério de Almeida

Corresponding author – sandroal@usp.br

Av. Prof. Lineu Prestes, 580, Bloco 17 (11) 3091-3661
05508-000-São Paulo, SP – Brasil dact@usp.br
